# Supplementary material for: Content-rich biological network constructed by mining PubMed abstracts
Source: BMC Bioinformatics. 2004 Oct 8;5:147. doi: 10.1186/1471-2105-5-147 (PMC528731; doi:10.1186/1471-2105-5-147)
Supplement: Additional File 5 — The original Chilibot query results of the term "long-term potentiation (LTP)" and 22 other terms, limiting the latest references analyzed to the years 1990, 1995, 2000, and 2004. [file 1471-2105-5-147-S5.bz2 › chilibotAdditionalFile5/ltp2000/html/PKA_SYNAPTOTAGMIN.html]

 


 **PKA** and **SYNAPTOTAGMIN** 
  
Found 3 abstracts in PubMed,  **3 abstracts were retrieved and analyzed**.  


---

 Search Google  |
 PDF files only 
|  EDU domain only 

---

- Biochemistry, 1998   **Binary interactions of the SNARE proteins syntaxin 4, SNAP23, and VAMP 2 and their regulation by phosphorylation.** .
  The SNARE hypothesis proposes that synaptic vesicles dock at presynaptic membranes via interactions among the vesicular, integral membrane proteins VAMP vesicle associated membrane protein and **synaptotagmin** and the target membrane proteins SNAP25 synaptosome associated protein with an Mr of 25 kDa and syntaxin 1.
  Non neuronal cells express isoforms of these proteins, believed to mediate secretory vesicle docking and or fusion.
  Secretion in neuronal and non neuronal systems differs in time course, Ca2ion dependence, and regulatory input.
  It is not known whether the non neuronal protein isoforms form complexes akin to those of their neuronal counterparts.
  In this study, we defined the binding characteristics of three SNARE proteins SNAP23, VAMP 2, and syntaxin 4.
  Binary, saturable interactions among all three partners VAMP 2 syntaxin 4, VAMP 2 SNAP23, and SNAP23 syntaxin 4 were measured in vitro.
  Unlike its neuronal counterpart, SNAP23 did not potentiate VAMP 2 binding to its putative t SNARE partner, syntaxin 4.
  The susceptibility of SNARE proteins to phosphorylation by exogenous kinases and their impact on binary interactions were explored.
  Syntaxin 4 was efficiently phosphorylated by casein kinase II CKII and cAMP dependent protein kinase **PKA** incorporating 0.8 and 3.9 mol of phosphate mol of syntaxin 4, respectively , while syntaxin 1 was only strongly phosphorylated by CKII.
  Each of the syntaxin isoforms was weakly phosphorylated by protein kinase C PKC <0.05 mol of phosphate mol of syntaxin 4 .
  Importantly, **PKA** but not casein kinase II phosphorylation of syntaxin 4 disrupted its binding to SNAP23.
  We hypothesize that **PKA** may modulate syntaxin 4 dependent SNARE complex formation to regulate exocytosis in non neuronal cells.

  - Neuron, 1998   **Visualization of cyclic AMP regulated presynaptic activity at cerebellar granule cells.** .
    Adenylyl cyclase AC modulation of vesicular cycling was visualized at cultured cerebellar granule cell synapses using the sequential uptake of antibodies directed against the intraluminal domain of **synaptotagmin** I.
    Vesicle recycling due to spontaneous transmitter release in the absence of action potentials was increased by the AC protein kinase A **PKA** activators forskolin and CPT cAMP.
    These effects were blocked by the **PKA** inhibitor Rp cAMPs.
    Cyclic AMP elevation also induced new cycling at previously silent sites.
    Activation of L AP4 sensitive mGluR reduced the cAMP **PKA** enhancement at preexisting synapses downstream of both AC and calcium channels.
    Modulation of the turnover and the number of vesicular release sites provide one mechanism that may underlie cAMP dependent cerebellar long term potentiation.

    - Proc Natl Acad Sci U S A, 1996   **Phosphorylation of synaptic vesicle proteins modulation of the alpha SNAP interaction with the core complex.** .
      We analyzed whether synaptic membrane trafficking proteins are substrates for casein kinase II, calcium calmodulin dependent protein kinase II, and cAMP dependent protein kinase **PKA** , three kinases implicated in the modulation of synaptic transmission.
      Each kinase phosphorylates a specific set of the vesicle proteins syntaxin 1A, N ethylmaleimide sensitive factor NSF , vesicle associated membrane protein VAMP , synaptosome associated 25 kDa protein SNAP 25 , n sec1, alpha soluble NSF attachment protein alpha SNAP , and **synaptotagmin**.
      VAMP is phosphorylated by calcium calmodulin dependent protein kinase II on serine 61.
      alpha SNAP is phosphorylated by **PKA**.
      the beta SNAP isoform is phosphorylated only 20% as efficiently.
      alpha SNAP phosphorylated by **PKA** binds to the core docking and fusion complex 10 times weaker than the dephosphorylated form.
      These studies provide a first glimpse at regulatory events that may be important in modulating neurotransmitter release during learning and memory.
